# Metabolomics, Transcriptome and Single-Cell RNA Sequencing Analysis of the Metabolic Heterogeneity between Oral Cancer Stem Cells and Differentiated Cancer Cells

**DOI:** 10.3390/cancers16020237

**Published:** 2024-01-05

**Authors:** Yuwen Miao, Pan Wang, Jinyan Huang, Xin Qi, Yingjiqiong Liang, Wenquan Zhao, Huiming Wang, Jiong Lyu, Huiyong Zhu

**Affiliations:** 1 Stomatology Hospital, School of Stomatology, Zhejiang University School of Medicine, Zhejiang Provincial Clinical Research Center for Oral Diseases, Key Laboratory of Oral Biomedical Research of Zhejiang Province, Cancer Center of Zhejiang University, Engineering Research Center of Oral Biomaterials and Devices of Zhejiang Province, Hangzhou 310020, China; whmy_1107@163.com; 2 Department of Stomatology, The First Affiliated Hospital, Zhejiang University School of Medicine, Hangzhou 310003, China; wangpan661@126.com (P.W.); zhuhuiyong@zju.edu.cn (H.Z.); 3 Biomedical Big Data Center, The First Affiliated Hospital, Zhejiang University School of Medicine, Hangzhou 310003, China

**Keywords:** oral cancer, cancer stem cell, metabolic heterogeneity, metabolomics, single-cell RNA sequencing

## Abstract

**Simple Summary:**

Cancer stem cell have certain metabolic properties that are distinct from their differentiated counterparts. Our aim is to characterize CSC metabolism in oral cancer. Our study have several impacts: 1. Previous metabolic studies of CSC mainly focused on few energy metabolism pathway. Here we used novel transcription/metabolic joint analysis to reveal comprehensive metabolic alteration of CSC in oral cancer. 2. Assessing CSC metabolic phenotype in vivo is challenging. Here We used single-cell sequencing to explore the metabolic characteristics of CSC in vivo. 3. Our data suggested oral CSCs are metabolically inactive compared with differentiated cancer cells. This state may allow CSCs to resist the metabolic therapeutic strategies currently used for highly proliferative tumors. This knowledge may allow us to better develop metabolic therapy against CSC in oral cancer.

**Abstract:**

Understanding the distinct metabolic characteristics of cancer stem cells (CSC) may allow us to better cope with the clinical challenges associated with them. In this study, OSCC cell lines (CAL27 and HSC3) and multicellular tumor spheroid (MCTS) models were used to generate CSC-like cells. Quasi-targeted metabolomics and RNA sequencing were used to explore altered metabolites and metabolism-related genes. Pathview was used to display the metabolites and transcriptome data in a KEGG pathway. The single-cell RNA sequencing data of six patients with oral cancer were analyzed to characterize in vivo CSC metabolism. The results showed that 19 metabolites (phosphoethanolamine, carbamoylphosphate, etc.) were upregulated and 109 metabolites (2-aminooctanoic acid, 7-ketocholesterol, etc.) were downregulated in both MCTS cells. Integration pathway analysis revealed altered activity in energy production (glycolysis, citric cycle, fatty acid oxidation), macromolecular synthesis (purine/pyrimidine metabolism, glycerophospholipids metabolism) and redox control (glutathione metabolism). Single-cell RNA sequencing analysis confirmed altered glycolysis, glutathione and glycerophospholipid metabolism in in vivo CSC. We concluded that CSCs are metabolically inactive compared with differentiated cancer cells. Thus, oral CSCs may resist current metabolic-related drugs. Our result may be helpful in developing better therapeutic strategies against CSC.

## 1. Introduction

Oral cancer is the term for malignancies developing in the oral cavity, and its risk factors include smoking, tobacco and alcohol [1]. Oral cancer has the 11th-highest incidence rate among all cancers [2]. Each year from 2010 to 2019, the incidence increased by about 1% and the mortality rate increased by 0.4% [3]. Oral squamous cell carcinoma (OSCC) accounts for about 90% of all oral cancers. Despite recent advances in imaging, surgery, radiation and systemic therapies, the overall survival of patients with OSCC has improved by only 5% in the last 20 years. The main reasons for treatment failure are local recurrence and lymph node metastasis [4,5].

Cancer cells reprogram their metabolism to meet the energy and anabolic requirements for survival and proliferation. Warburg [6] first observed that even when adequate oxygen is available, cancer cells preferentially perform glycolysis rather than oxidative phosphorylation. Recent studies have shown that metabolites are not only essential for the cellular structure and energy generation but also serve critical functions as signaling molecules, immune modulators, endogenous toxins and environmental sensors [7]. Natalya [8] outlined several key aspects of cancer metabolism, such as dysregulated glucose and amino acid uptake, a heightened demand for electron acceptors and metabolic interactions within the tumor microenvironment. These distinct metabolic traits associated with cancer offer insights into tumor imaging, prognostic assessments and potential therapeutic approaches [9].

Understanding cancer metabolism has implications for clinical oncology. Researchers have recently begun to pay more attention to intratumoral metabolic heterogeneity. A typical example is the metabolic heterogeneity between cancer stem cells (CSCs) and normal tumor cells. According to CSC theory, CSCs are a distinct population of cancer cells that have a high self-renewal ability and tumorigenic potential, and these characteristics play an important role in cancer relapse, metastasis and radiotherapy/chemotherapy resistance [10,11]. CSCs were first identified in patients with leukemia and then in many solid tumors, including OSCC [12,13,14,15]. Recent evidence suggests that CSC populations are in a dynamic and plastic state [16,17].

CSCs may have certain metabolic properties distinct from their differentiated counterparts [18]. Research in the fields of liver cancer, glioma, breast cancer and other types of cancer has elucidated these properties [19,20]. Understanding the distinct metabolic characteristics of CSCs may allow us to better cope with the clinical challenges associated with them. Our research team is interested in the metabolic characteristics of oral CSCs, which are currently unknown. In this study, we used OSCC cell lines and multicellular tumor spheroid (MCTS) models to generate CSC-like cells. We then used quasi-targeted metabolomics to quantify thousands of metabolites and performed RNA sequencing (RNA-seq) to analyze the transcriptional level of metabolic genes. The altered metabolites and genes were integrated into a Kyoto Encyclopedia of Genes and Genomes (KEGG) pathway analysis. In addition, we performed a single-cell transcriptome analysis of six OSCC tumors to preliminarily explore the metabolic phenotypes of oral CSCs in their native microenvironments in humans.

## 2. Materials and Methods

### 2.1. Cells and Culture Conditions

Experiments using HSC3 and CAL27 cell lines were maintained by the Laboratory of Stomatology, Zhejiang University. Both cell lines were cultured in Dulbecco’s modified Eagle’s medium/Nutrient Mixture F-12 (DMEM/F-12, Cellmax, Beijing, China) containing 10% fetal bovine serum and incubated at 37 °C in humidified air with 5% carbon dioxide.

### 2.2. MCTS Models

MCTS models were used to enrich CSCs in the HSC3 and CAL27 cell lines, as described in a previous report [21]. Briefly, when the adherent cells reached 85–90% confluence, they were dissociated into a single-cell suspension and resuspended in serum-free DMEM/F-12 supplemented with 2% B-27 (Gibco, Waltham, MA, USA), 10 ng/mL epidermal growth factor (EGF) (PeproTech, Cranbury, NJ, USA) and 10 ng/mL basic fibroblast growth factor (bFGF) (PeproTech) at a cell density of 1 × 10^5^ cells/mL in a poly(2-hydroxyethyl methacrylate)-coated plate [22,23]. The culture medium was refreshed every 3 days. On day 7, the MCTS models were used for further testing and analysis.

### 2.3. Reverse Transcription Quantitative Polymerase Chain Reaction (RT-qPCR)

Total cellular RNA was extracted using a Universal RNA Extraction Kit (Takara, Beijing, China) according to the manufacturer’s instructions. The mRNA was reverse-transcribed using a PrimeScript IV 1st strand cDNA Synthesis Mix (Takara). Then, RT-qPCR of the cDNA was performed using a TB Green Premix Ex Taq™ II FAST qPCR (Takara) on a ViiA7 System (Thermo Fisher Scientific, Waltham, MA, USA). The primer sequences used in this study were designed at the NCBI website (https://www.ncbi.nlm.nih.gov/, accessed on 10 April 2023) and were as follows: *SOX2* (forward, 5′-AACTCCATGACCAGCTCGCAGA-3′ and reverse, 5′-GGACTTGACCACCGAACCCAT-3′), *NANOG* (forward, 5′-TGGCTCTGTTTTGCTATATCCC-3′ and reverse, 5′-CATTACGATGCAGCAAATACGAGA-3′), *OCT4* (forward, 5′-TATGCAAAGCAGAAACCCTCGT-3′ and reverse, 5′-TTCTCCAGGTTGCCTCTCACTCG-3′) and *GAPDH* or actin (forward, 5′-GGAGCGAGATCCCTCCAAAAT-3′ and reverse, 5′-GGCTGTTGTCATACTTCTCATGG-3′).

### 2.4. Flow Cytometry

The collected cells were dissociated and resuspended into a single-cell suspension. Fluorescence-conjugated CD133 antibody (ab252126, diluted to 1:1000; Abcam, Cambridge, UK) was added, and the samples were incubated for 30 min on ice. The proportions of CD133+ cells were determined using a cytometer (CytoFLEX; Beckman Coulter Life Sciences, Brea, CA, USA).

### 2.5. Sphere-Forming Assays

The experimental procedure for the sphere-forming assays was similar to that for the MCTS models; the main difference was that the single-cell suspension was seeded at a density of 2000 cells and the serum-free DMEM/F-12 medium contained 20 ng/mL EGF, 20 ng/mL bFGF and 2% B-27 [24]. The medium was refreshed every 2–3 days. Seven days later, spheres larger than 50 µm were counted using an inverted microscope.

### 2.6. Transcriptome Sequencing

For transcriptome sequencing, all samples were processed in triplicate. A total of 1 × 10^6^ adherent cells or multicellular sphere cells were collected and sent to a commercialized testing company (Novogene, Tianjin, China) for transcriptome sequencing. The experimental procedures and subsequent data processing of transcriptome sequencing are provided in the Supplementary Materials and Methods (Method S1). Briefly, after library preparation, low-quality bases were removed from raw data, and Hisat2 (v2.0.5) [25] was used to map reads to the reference genome. FeatureCounts (v1.5.0-p3) [26] was used to count the reads numbers mapped to each gene; then, the FPKM of each gene was calculated. The differential expression analysis of experiment/control groups was performed using the DESeq2 R package (1.20.0) [27]. A corrected *p*-value of 0.05 and absolute fold-change of 1.5 were set as the threshold for significantly differential expression. The KEGG enrichment analysis of differentially expressed genes was implemented by the clusterProfiler R package (3.8.1) [28].

### 2.7. Quasi-Targeted Metabolomics

For quasi-targeted metabolomics, all samples were processed in quintuplicate. A total of 5 × 10^6^ adherent cells or multicellular sphere cells were seeded at 2.5 × 10^5^/mL in DMEM/F-12 without fetal bovine serum or B-27/EGF/bFGF for 24 h. All cells were then collected and sent to a commercial testing company (Novogene) for quasi-targeted metabolomics. The experimental procedures and subsequent data processing of quasi-targeted metabolomics are provided in the Supplementary Materials and Methods (Method S2). Briefly, the metabolomics samples were injected into the LC-MS/MS system (ExionLC™ AD system (SCIEX) coupled with a QTRAP^®^ 6500+ mass spectrometer (SCIEX)). The detection of the experimental samples using MRM (Multiple Reaction Monitoring) were based on the novogene in-house database. The data files generated by HPLC-MS/MS were processed using SCIEX OS Version 1.4 to integrate and correct the peak. These metabolites were annotated using the KEGG database (http://www.genome.jp/kegg/, accessed on 13 November 2022), HMDB database (http://www.hmdb.ca/, accessed on 13 November 2022) and Lipidmaps database (http://www.lipidmaps.org/, accessed on 13 November 2022). We applied univariate analysis (*t*-test) to calculate the statistical significance (*p*-value). The metabolites with a VIP > 1, a *p*-value< 0.05 and a fold change > 1.5 were considered to be differential metabolites.

### 2.8. Single-Cell RNA Sequencing Analysis

The single-cell RNA sequencing data of tumor samples from six patients with oral cancer were downloaded from the Gene Expression Omnibus database (accession number GSE172577) [29]. Seurat (Version 4.3.0) [30] was used for downstream analysis. The data analysis was performed as follows: (1) the data of the six samples were integrated after being filtered by the minimum number of cells (*n* = 3), a feature count of 200–6000 and a mitochondrial gene proportion of <0.05. (2) We employ a global-scaling normalization method to normalizes the feature expression measurements for each cell. (3) After applying linear transformation to scale the data, PCA was performed on the scaled data. (4) A graph-based clustering approach was used to cluster the cells, and non-linear dimensional reduction (UMAP) was performed to visualize and explore these datasets. (5) After differential expressed features were analyzed, cluster definition and annotation were performed by SingleR (Version 2.2.0) [31] combined with manual annotation according to existing knowledge. Fourth, the FindMarkers function was used to identify differentially expressed genes (fold change of >1.5 and adjusted *p*-value of <0.05) between CSC clusters and non-CSC clusters.

### 2.9. Survival Analysis

A survival analysis was performed using the R package “survival” (version 3.5-7). The mRNA expression data (Z-score) and relevant clinical information of 528 samples of head and neck squamous cell carcinoma (The Cancer Genome Atlas of the Pan-Cancer Atlas) were downloaded from the cBioPortal website (http://www.cbioportal.org/, accessed on 28 June 2023). For each gene analyzed, samples with a Z-score above the median were defined as exhibiting a high expression, and those with a Z-score below the median were defined as exhibiting a low expression.

## 3. Results

### 3.1. MCTS Increases the Stemness of Tumor Cells

**Figure 1:**
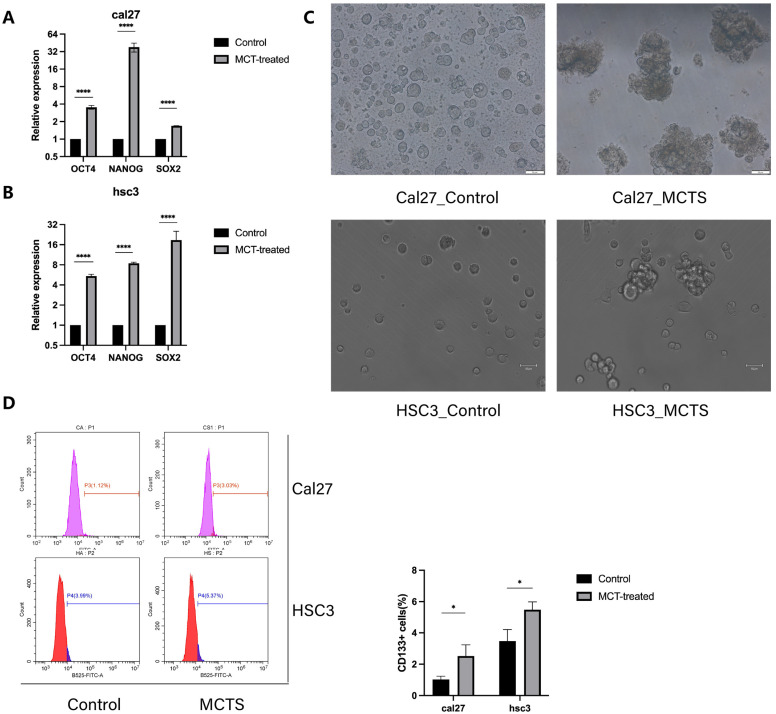
MCTs increase the stemness of tumor cells. (**A**,**B**) Relative mRNA levels of the stemness-related gene, as determined by qRT-PCR. Data are given as relative expression levels (n = 3, **** *p* < 0.0001, *t*-test) (**C**) Sphere formation assay of control- and MCT-treated cells in cal27 and hsc3 (n = 3). (**D**) FACS strategy for sorting CD133+ cells and the percentage of CD133+ cells (n = 3, * *p* < 0.05, *t*-test).

Metabolomics research requires a very large number of cells. Because of the very low proportion of CSCs (<3%), several commonly used methods for isolating CSCs (e.g., flow cytometry sorting by CSC markers [32] or spheroid colony formation [33]) seem unacceptable from the viewpoints of cost and labor. Therefore, we employed MCTS models [21,34] to obtain an adequate number of cells with CSC properties. To validate the changes in stemness of the tumor cells processed by the MCTS model, we evaluated the changes in transcription factors (*OCT4*, *NANOG*, and *SOX2*) using qRT-PCR. As shown in Figure 1, the expression of these genes in tumor cells processed by the MCTS model increased significantly (Figure 1A,B). The sphere-forming assays suggested that the MCTS cells had an increased sphere-forming ability compared with the adherent cancer cells (Figure 1C). We subsequently used flow cytometry (with the CSC surface marker CD133) to evaluate the changes in the proportion of CSCs. After processing with the MCTS model, the proportion of CD133+ cells in both the CAL27 and HSC3 cell lines increased significantly (Figure 1D). These results indicate that, compared with adherent cells, MCTS cells have significantly enhanced stemness.

### 3.2. Metabolomics and Transcriptome Results

**Figure 2:**
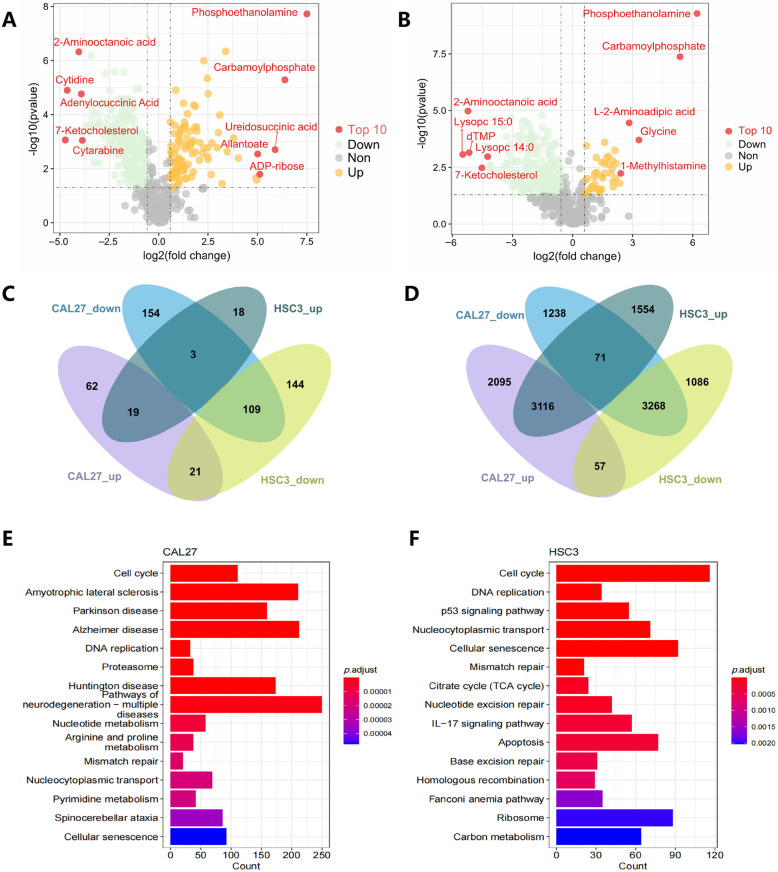
(**A**,**B**), Volcano plots of differential metabolites between the control and MCTS, in CAL27 (**A**) and HSC3 (**B**). The yellow point, green point and grey point indicate the metabolites that were significantly upregulated, significantly downregulated and non-significantly different, respectively. The top ten metabolites with the greatest difference in expression between the control and MCTS are annotated in a red color. (**C**,**D**) Venn diagrams show the number of metabolites (**C**) and genes (**D**) that are significantly increased or decreased in CAL27 and HSC3 cells. (**E**,**F**) KEGG pathway enrichment of differentiated expressed genes between the control and MCTS in CAL27 (**E**) and HSC3 (**F**).

Metabolomics technologies allow us to identify and quantify hundreds of metabolites in biological samples [35]. In this study, we conducted quasi-targeted metabolomics, a method that offers higher quantitative precision than untargeted metabolomics. Our objective was to quantify alterations in intracellular metabolites within OSCC cell lines, specifically MCTS. The sample details are present in Table S1. Our quasi-targeted metabolomics procedures were based on the Novogene database, which includes 2200 metabolites. The results are shown in Table S2. Finally, 368 metabolites (upregulated, n = 102; downregulated, n = 266) were found to be significantly changed in the CAL27 cells (Figure 2A), and 314 metabolites (upregulated, n = 40; downregulated, n = 274) were found to be significantly changed between the control and MCTS in HSC3 cells (Figure 2B).

We also performed transcriptome sequencing to explore global gene alterations after the MCTS process. We found a total of 4743 upregulated and 4412 downregulated genes in HSC3 MCTS and 5269 upregulated and 4578 downregulated genes in CAL27 cells. The KEGG pathway enrichment analysis (Figure 2E,F) shows that differential expressed genes were enriched in the cell cycle, DNA replication and cell senescence, suggesting a state of cell senescence.

The Venn diagram shows that 19/109 metabolites were upregulated or downregulated in both HSC and CAL27 MCTS (Figure 2C), and 3116/3268 genes were upregulated or downregulated in these cells (Figure 2D). These metabolites and genes were selected for subsequent analysis.

### 3.3. Integration of Metabolomic and Transcriptome Pathway Analysis

Pathview (https://pathview.uncc.edu/, accessed on 18 June 2023) [36] was used to display the metabolites and transcriptome data in KEGG pathway graphs. We mainly focused our analysis on the metabolic properties of energy production, macromolecular synthesis and redox control in cancers [37].

#### 3.3.1. Energy Production

Glucose metabolism is the main pathway by which tumor cells obtain energy. In glycolysis, we found that fructose 6-phosphate, glucose 6-phosphate and fructose 1,6-bisphosphate were upregulated in MCTS, while lactic acid was downregulated. Some genes involved in glycolysis were upregulated, whereas others were downregulated (Figure S1). The expression of *HK2*, a key enzyme for glycolysis, was significantly high. In the citric cycle, cis-aconitic acid and succinic acid were downregulated, and most genes in this pathway were also downregulated (Figure S1). Fatty acid oxidation (FAO) is another energy source for cancer cells. We found that carnitines (decanoylcarnitine, isovalerylcarnitine and hexanoylcarnitine) were upregulated in MCTS. A marked decline in FAO-related genes was observed (Figure S1). *CPT1A*, the key enzyme of FAO, was also significantly downregulated in MCTS.

#### 3.3.2. Macromolecular Synthesis

Purines and pyrimidines are important building blocks for proliferating cells. We observed the significant downregulation of metabolites (guanine, adenosine, dTMP, UMP and others) and decreased expression levels of several enzymes involved in purine/pyrimidine metabolism (Figure S1). Glycerophospholipids are involved in the composition of the cell membrane. In the present study, phosphoethanolamine was the most significantly elevated metabolite in CSCs, while lysophospholipids (lysophosphatidylethanolamine and lysophosphatidylcholine) were significantly downregulated in CSCs. Pathway analysis showed that several enzymes related to glycerophospholipid synthesis/degradation were altered in MCTS (Figure S1).

#### 3.3.3. Redox Control and Other Pathways

The glutathione (GSH) system is an important cellular antioxidant defense mechanism. We found decreased levels of both GSH and oxidized GSH in CSCs. The expression of several related enzymes was found to be upregulated or downregulated in CSCs (Figure S1). *GPX4*, a key molecule for maintaining redox homeostasis, was upregulated. Besides the above-mentioned energy production, macromolecular synthesis and redox control pathways, there were also many altered metabolites involved in known or unknown related pathways, such as carbamoyl phosphate, 1-methylhistamine and adipic acid, among others.

### 3.4. Alteration of Metabolism Genes in Clinical Samples

The metabolic heterogeneity of cancer results from a complex set of factors [37]. Our cell line data may reflect these metabolic alterations by showing intrinsic molecular heterogeneity between CSCs and differentiated cancer cells. In vivo, however, CSCs reside in a complex tumor microenvironment that impacts tumor metabolism. Here, we conducted a single-cell transcriptomic sequencing analysis of clinical samples to characterize in vivo CSC metabolism.

**Figure 3:**
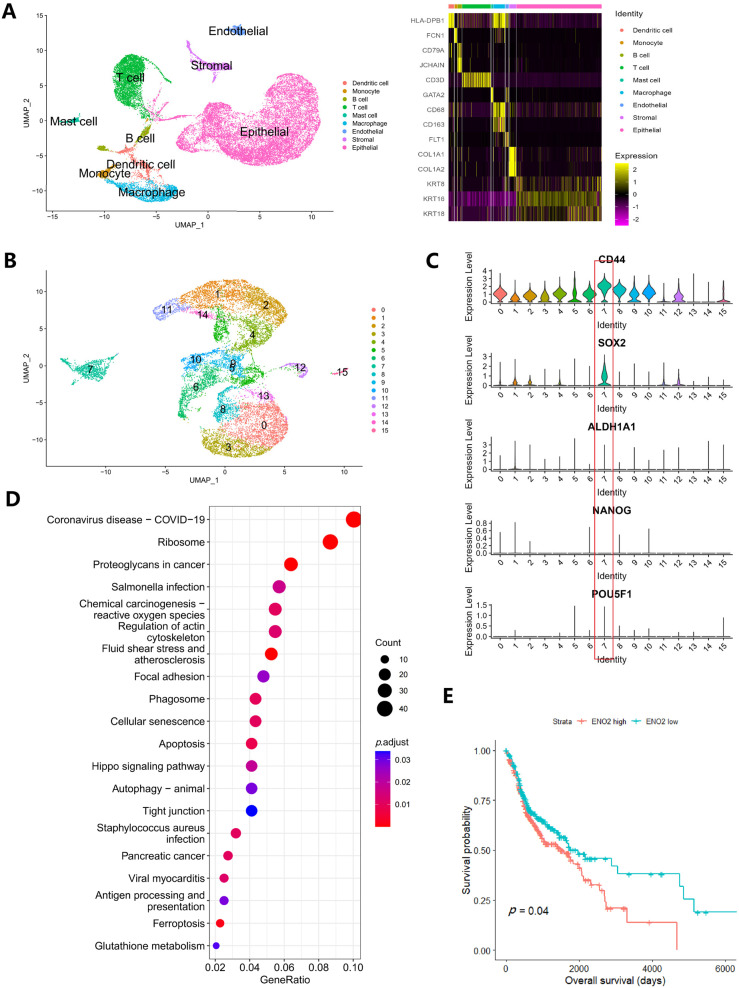
(**A**) UMAP of 25,531 cells in oral cancer and heatmap for marker genes for each cell group; (**B**) UMAP of 14,484 cancer cells revealed 16 clusters, and violin plots of CSC markers (**C**) suggest highest expression of CD44, SOX2 in cluster 7; (**D**) KEGG pathway enrichment of differentiated expressed genes in the CSC cluster; (**E**) Survival analysis revealed that high ENO2 expression is associated with poor survival.

In total, 25,531 cells were entered into the analysis. After data processing, 23 cell clusters were identified (Figure 3A), and 10 of these clusters were annotated as epithelial cells or keratinocytes. The data of these 10 clusters (including 14,484 cells) were extracted separately and subjected to repeat data processing and clustering. Finally, 16 clusters were identified (Figure 3B). In previous studies, *CD44*, *ALDH1A1*, *SOX2*, *NANOG* and *POU5F1* were regarded as CSC markers. Here, our violin plots showed that *SOX2* was mainly enriched in cluster 7 and that cluster 7 also had the highest *CD44* expression, indicating that cluster 7 was the CSC group (Figure 3C). We then regrouped the tumor cells into a CSC group (cluster 7) and a non-CSC group (other clusters). Finally, 864 genes were found to be differentially expressed in CSCs (Table S3). These genes underwent KEGG pathway enrichment analysis (Figure 3D).

Next, we focused on the following pathways: glycolysis, citrate cycle, fatty acid degradation, GSH metabolism and glycerophospholipid metabolism. The result suggested active glycolysis, GSH and glycerophospholipid metabolism (Figure S2), while few genes in the citrate cycle or fatty acid degradation pathway were detected. Thirteen common differentially expressed genes were identified in these pathways. Nine of them (*ENO2*, *HK2*, *GPI*, *GPX3*, *GPX4*, *ODC1*, *GPCPD1*, *PLD1* and *PLPP2*) showed consistent tendencies in both the cell lines and clinical samples. Because the metabolic phenotype of CSCs may have prognostic value, we performed a survival analysis of these nine genes. The result showed that *ENO2* had prognostic value; the median survival time for patients with higher *ENO2* expression is 50.1 months (31.5–68.7 months), compared to 65.7 months (34.6–96.8 months) in those with lower *ENO2* expression (Figure 3E).

## 4. Discussion

Divergent metabolic phenotypes reflect the molecular heterogeneity of cancer cells and inconsistencies in the microenvironment. Greater knowledge of the heterogeneous metabolic nature of cancer can enable the specific targeting of subclones in a single tumor. To our knowledge, this is the first metabolomics study to characterize CSC metabolism in oral cancer. Metabolomics is routinely applied as a tool for biomarker discovery. With the recent advances in metabolomics technology and bioinformatic tools, metabolomics can not only establish correlations between altered metabolites and particular cell/disease phenotypes but can also help us understand the role of these metabolic alterations in phenotypic outcomes and thus guide novel therapy [38,39]. The use of a multilayered omics strategy, such as the integration of metabolomics with transcriptomics or proteomics, can help to determine the relationships between gene/protein expression and metabolites, as well as the balance between metabolite production and consumption [40]. In the present study, we used an integrated metabolomics/transcriptomics approach to explore the metabolic heterogeneity between CSCs and normal oral cancer cells, and our results showed a comprehensive stemness-related metabolic phenotype.

During the past few decades, the metabolic phenotype of CSCs has been a subject of in-depth research. Most studies to date have focused on the energy metabolism of CSCs. With respect to glucose metabolism, CSCs have been described as glycolytic or reliant on oxidative phosphorylation in a tumor type-dependent manner [18,41,42]. With respect to lipid metabolism, some studies have suggested that CSCs rely on FAO because the inhibition of FAO can reduce the number of CSCs in patients with leukemia and liver cancer [43,44]. In the present study, CSCs showed a decrease in metabolites and genes in the citric acid cycle, along with the overexpression of several glycolytic genes. This suggests that oral CSCs rely more on glycolytic than oxidative phosphorylation, which is similar to another type of head and neck cancer: nasopharyngeal cancer [45,46]. Our results also suggest decreased FAO activity in oral cancer CSCs, indicating that CSCs obtain less energy from fatty acids than differentiated cancer cells do.

Studies of CSC metabolism focusing on topics beyond energy metabolism are rare. Here, we found decreased GSH and oxidized GSH but upregulated *GPX4* expression in MCTS. We believe that the CSCs reached redox homeostasis with lower GSH levels because of reduced mitochondrial metabolism; however, the antioxidant capacity was still sufficient to overcome potential oxidative stress. Some anabolism pathways are also downregulated in CSCs, such as purine/pyrimidine metabolism and one-carbon metabolism, which may explain the weak proliferative characteristics of CSCs (CSC senescence) [47,48]. In addition, we found that some metabolism pathways or metabolites were significantly altered in MCTS (e.g., glycerophospholipid metabolism). These metabolites are rarely studied in cancers. Determining whether these alterations have any effect on the properties of CSCs requires further validation in experimental models.

According to a review by Heiden [9], the reprogrammed metabolic activities of cancer can be classified as transforming activities, enabling activities and neutral activities. The author suggested that transforming and enabling metabolic activities are therapeutic targets, whereas neutral metabolic activities may act as diagnostic or predictive biomarkers. Most existing and investigational metabolic therapies work against enabling metabolic activities, which support the survival, proliferation and progression of cancers (e.g., drugs targeting glycolysis, glutamine metabolism or nucleic acid synthesis) [49,50]. Based on our data, we believe that oral CSCs are not only in a state of senescence but are also metabolically inactive compared with differentiated cancer cells. This state may allow CSCs to resist the metabolic therapeutic strategies currently used for highly proliferative tumors. Other metabolic strategies should be explored to overcome the relapse/metastasis caused by CSCs.

A thorough understanding of the metabolic reprogramming of oral CSCs is an important step to developing better therapeutic strategies against this therapy-resistant cell population. However, there is still a large gap between obtaining metabolomics results and developing novel treatments. In some cases, altered metabolism may result in unique metabolic dependencies. Identifying the metabolic vulnerabilities of CSCs by experimental models may aid the development of strategies that inhibit or eliminate CSCs. Additionally, some metabolites or enzymes may participate in cellular signaling and affect cell functions or phenotypes, as well as play a role in metabolic reactions [50,51,52]. If metabolites or metabolic enzymes are found to participate in the cell signaling involved in stemness, the opportunity to induce the differentiation of CSCs (also called differentiation therapy) will arise. Combining differentiation therapy with conventional chemotherapy or radiotherapy may improve the effects of clinical therapy.

This study had some limitations. First, metabolite identification was incomplete due to limitations with the library, experimental sensitivity and metabolite extraction. Humans are estimated to have > 1 million metabolites [7,49]. Many metabolites are not identified in metabolomics studies because their concentrations are too low or they do not exist in the annotation library. The second limitation is that although our transcriptome sequencing reflects the mRNA expression of metabolic genes, metabolite levels are non-linearly dependent on enzyme abundance [40], and metabolic activity may also be regulated by other mechanisms (e.g., allosteric regulation). Therefore, the metabolic properties of CSCs may be more complex than indicated by the metabolomic/transcriptome results. Third, we used two OSCC cell lines in our experiment, while the results would be more reliable if more cell lines were used. Fourth, assessing the metabolic phenotypes of in vivo CSCs in their native microenvironment remains challenging because of limitations in isolating them and assessing their metabolism at the single-cell level. Our scRNA sequencing analysis is only the first step. Further progress in metabolic research technology may help us better understand the heterogeneity of cancer cells.

## 5. Conclusions

For the first time, we characterize comprehensive CSC metabolism in oral cancer. CSCs showed a state of senescence but are also metabolically inactive compared with differentiated cancer cells. Oral CSCs may resist current metabolic therapeutic strategies.

## Data Availability

Transcriptome data were uploaded to the Gene Expression Omnibus database (accession number GSE228899). For scRNA-seq, publicly available datasets were analyzed in this study. These data can be found in (GSE172577). Other data are available in supplementary files. The software code created for single-cell RNA sequencing analysis can be obtained at: https://github.com/Lyu9999/cancers_CSC_metabolic_scRNA.

## Supplementary Materials

The following supporting information can be downloaded at: https://www.mdpi.com/article/10.3390/cancers16020237/s1, Figure S1: Integrated metabolites and transcriptome analysis by Pathview; Figure S2: KEGG pathway analysis of ScRNA-seq data for certain metabolic pathways; Table S1: Detail of samples for metabolomics, transcriptome and single-cell RNA sequencing Analysis; Table S2: The metabolomics results; Table S3: Differentiated expressed genes between CSC and non-CSC revealed by scRNA-seq; Method S1: Procedure of transcriptome sequencing; Method S2: Procedure of quasi-targeted metabolomics.
