# Supplementary material for: Metabolomics, Transcriptome and Single-Cell RNA Sequencing Analysis of the Metabolic Heterogeneity between Oral Cancer Stem Cells and Differentiated Cancer Cells
Source: Cancers (Basel). 2024 Jan 5;16(2):237. doi: 10.3390/cancers16020237 (PMC10813553; doi:10.3390/cancers16020237)
Supplement: Supplementary file 1 [file cancers-16-00237-s001.zip › Supplementary figure S1.pdf]

**S.figure Glycolysis pathway (from integrated Metabolomics & transcriptome data )**

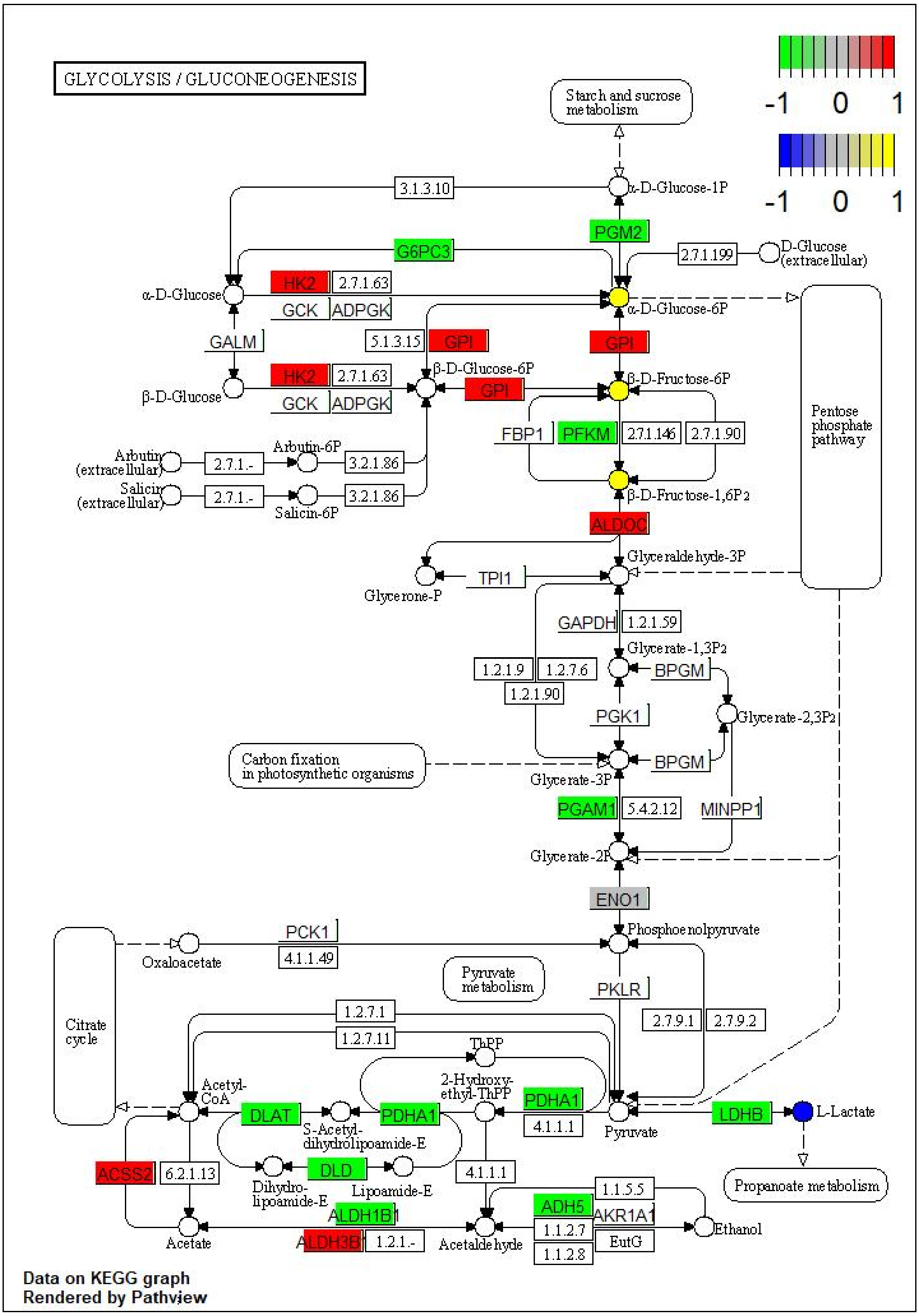


Figure: Transcription/metabolic joint analysis by Pathview revealed alterations of Glycolysis pathway in OSCC cell lines (MCTS-treated). The red box showed up-regulated genes, green box showed down-regulated genes. The yellow cycle showed up-regulated metabolites, and blue cycle showed down-regulated metabolites.

**S.figure Citrate cycle (from integrated Metabolomics & transcriptome data )**

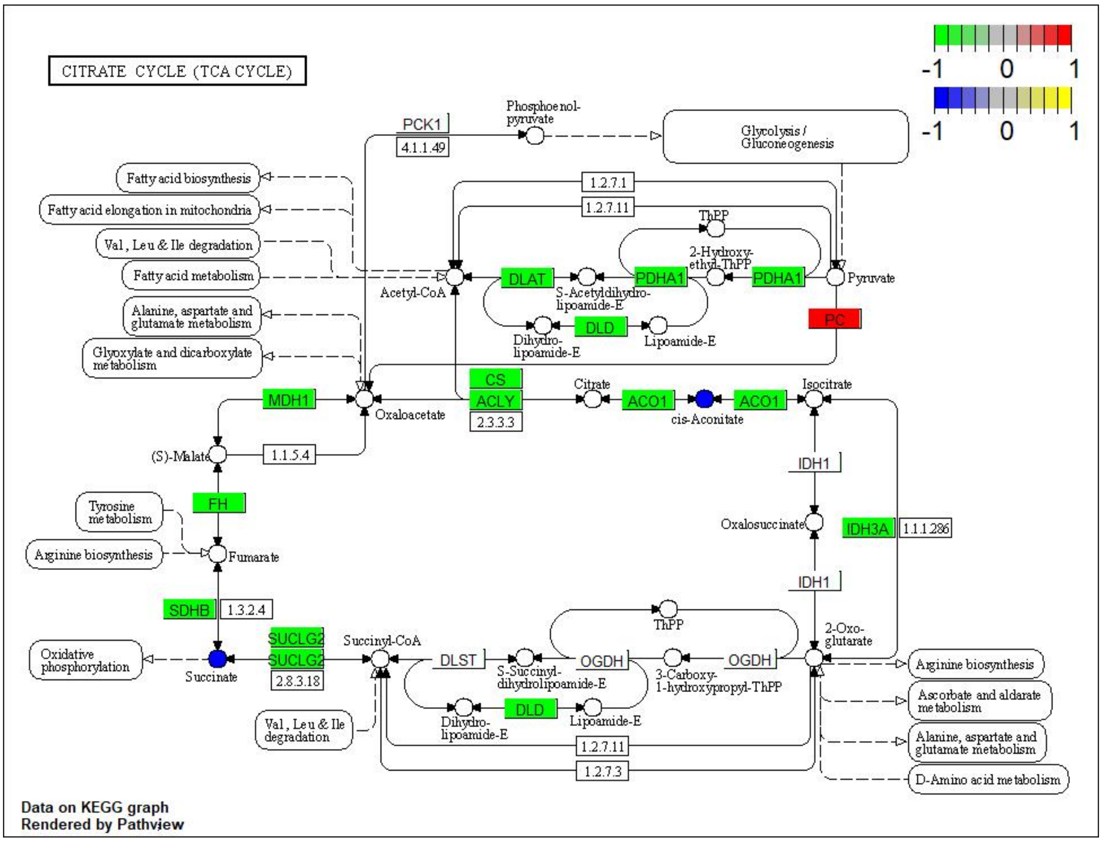


Figure: Transcription/metabolic joint analysis by Pathview revealed alterations of Citrate cycle in OSCC cell lines (MCTS-treated). The red box showed up-regulated genes, green box showed down-regulated genes. The yellow cycle showed up-regulated metabolites, and blue cycle showed down-regulated metabolites.

**S.figure Fatty acid degradation (from integrated Metabolomics & transcriptome data )**

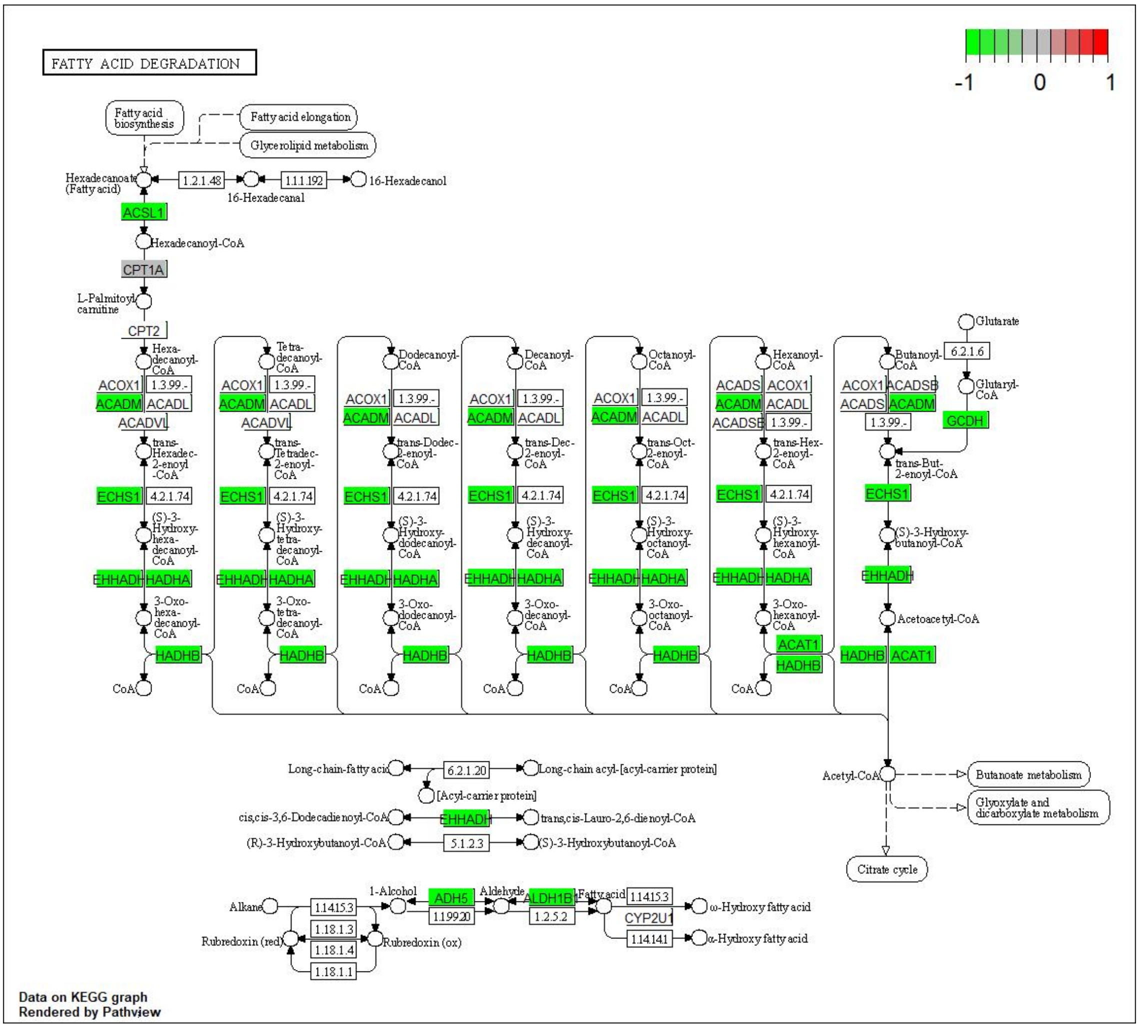


Figure: Transcription/metabolic joint analysis by Pathview revealed alterations of Fatty acid degradation in OSCC cell lines (MCTS-treated). The red box showed up-regulated genes, green box showed down-regulated genes. The yellow cycle showed up-regulated metabolites, and blue cycle showed down-regulated metabolites.

**S.figure Purine metabolism (from integrated Metabolomics & transcriptome data )**

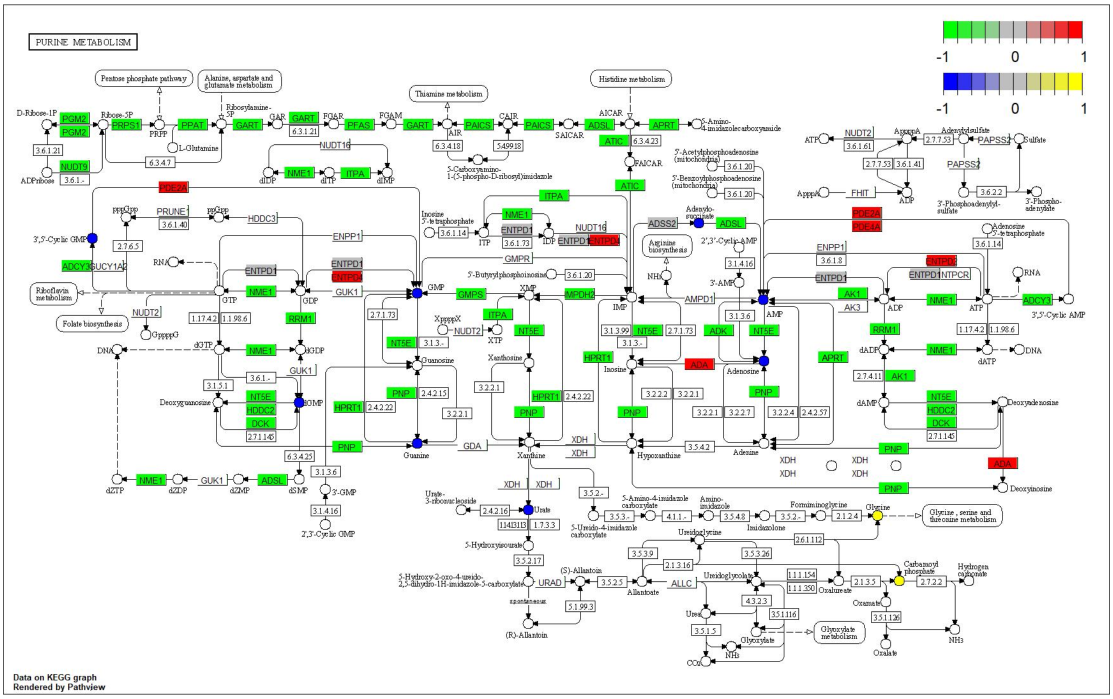


Figure: Transcription/metabolic joint analysis by Pathview revealed alterations of Purine metabolism in OSCC cell lines (MCTS-treated). The red box showed up-regulated genes, green box showed down-regulated genes. The yellow cycle showed up-regulated metabolites, and blue cycle showed down-regulated metabolites.

**S.figure Pyrimidine metabolism (from integrated Metabolomics & transcriptome data )**

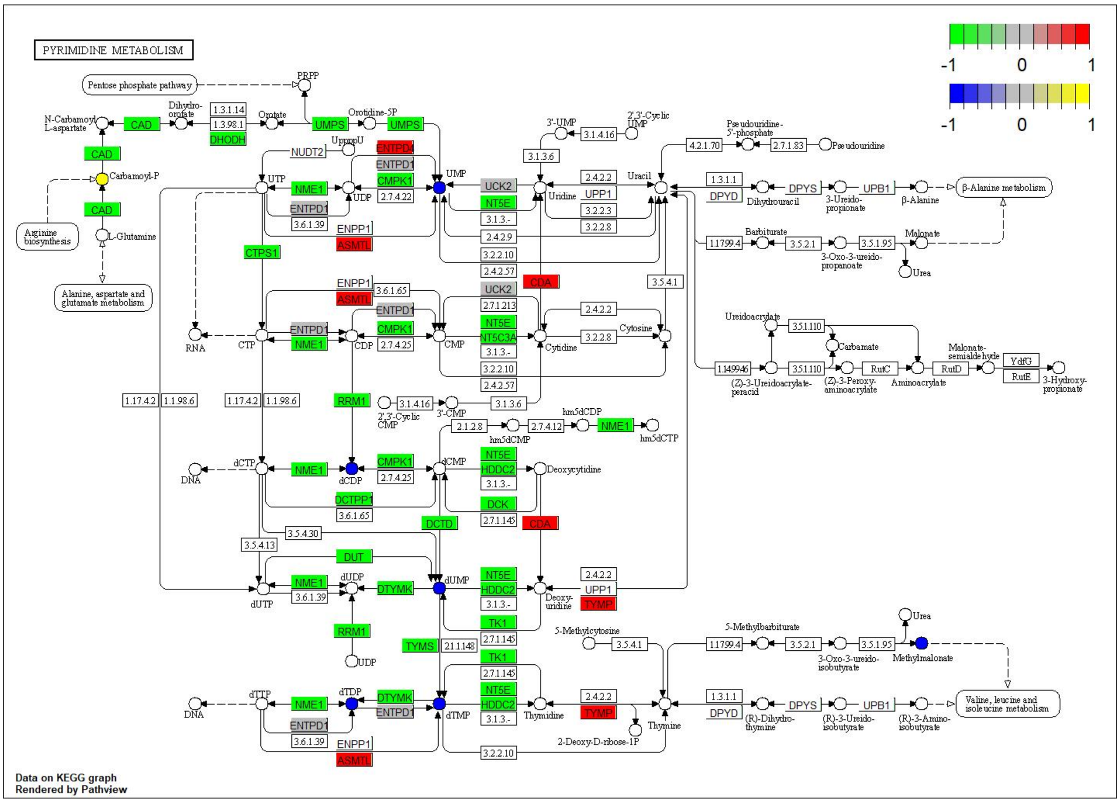


Figure: Transcription/metabolic joint analysis by Pathview revealed alterations of Pyrimidine metabolism in OSCC cell lines (MCTS-treated). The red box showed up-regulated genes, green box showed down-regulated genes. The yellow cycle showed up-regulated metabolites, and blue cycle showed down-regulated metabolites.

**S.figure Glutathione metabolism (from integrated Metabolomics & transcriptome data )**

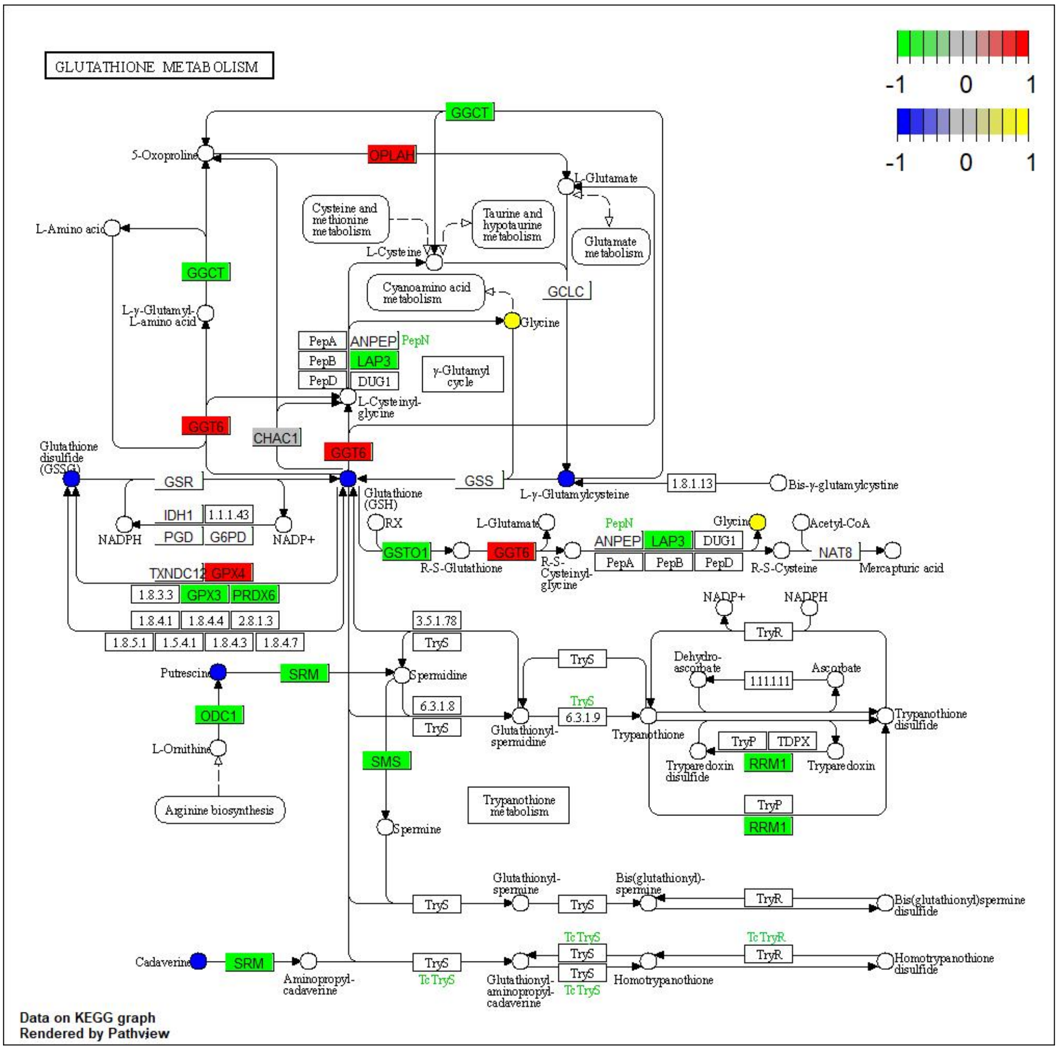


Figure: Transcription/metabolic joint analysis by Pathview revealed alterations of Glutathione metabolism in OSCC cell lines (MCTS-treated). The red box showed up-regulated genes, green box showed down-regulated genes. The yellow cycle showed up-regulated metabolites, and blue cycle showed down-regulated metabolites.

**S.figure Glycerophospholipid metabolism (from integrated Metabolomics & transcriptome data )**

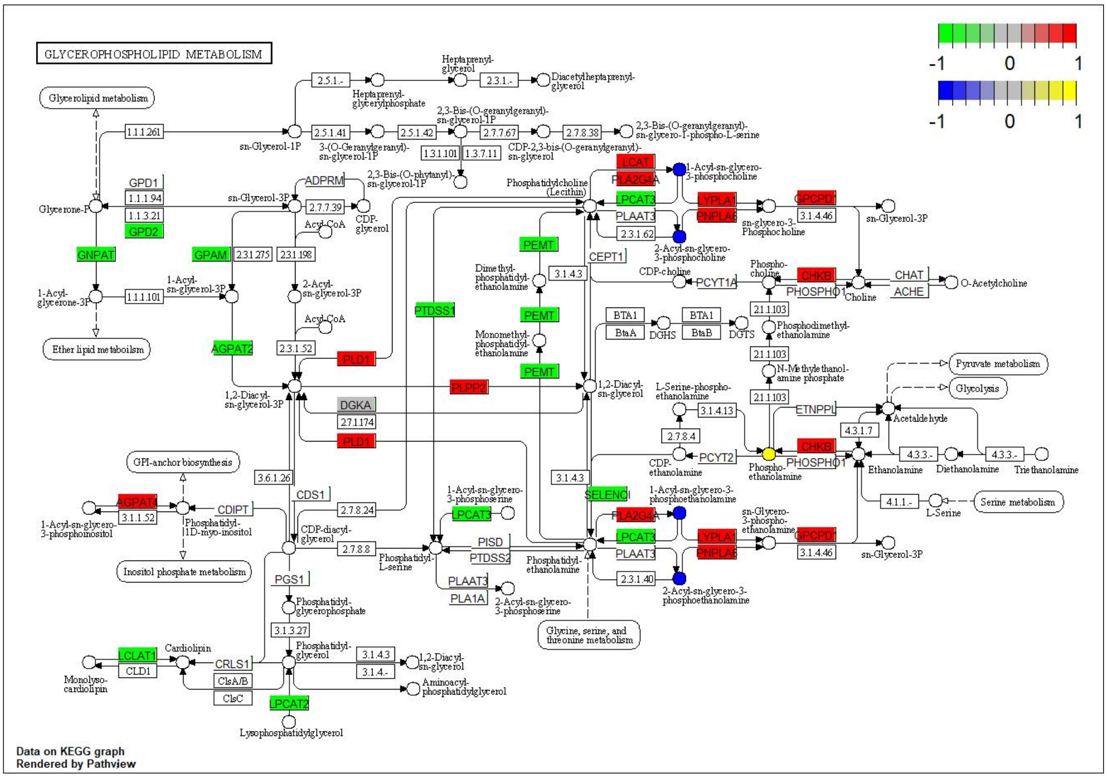


Figure: Transcription/metabolic joint analysis by Pathview revealed alterations of Glycerophospholipid metabolism in OSCC cell lines (MCTS-treated). The red box showed up-regulated genes, green box showed down-regulated genes. The yellow cycle showed up-regulated metabolites, and blue cycle showed down-regulated metabolites.
