# Supplementary material for: Metabolomics, Transcriptome and Single-Cell RNA Sequencing Analysis of the Metabolic Heterogeneity between Oral Cancer Stem Cells and Differentiated Cancer Cells
Source: Cancers (Basel). 2024 Jan 5;16(2):237. doi: 10.3390/cancers16020237 (PMC10813553; doi:10.3390/cancers16020237)
Supplement: Supplementary file 1 [file cancers-16-00237-s001.zip › supplementary figure S2.pdf]

S.figure Glycolysis metabolism (from scRNA-sequencing data)

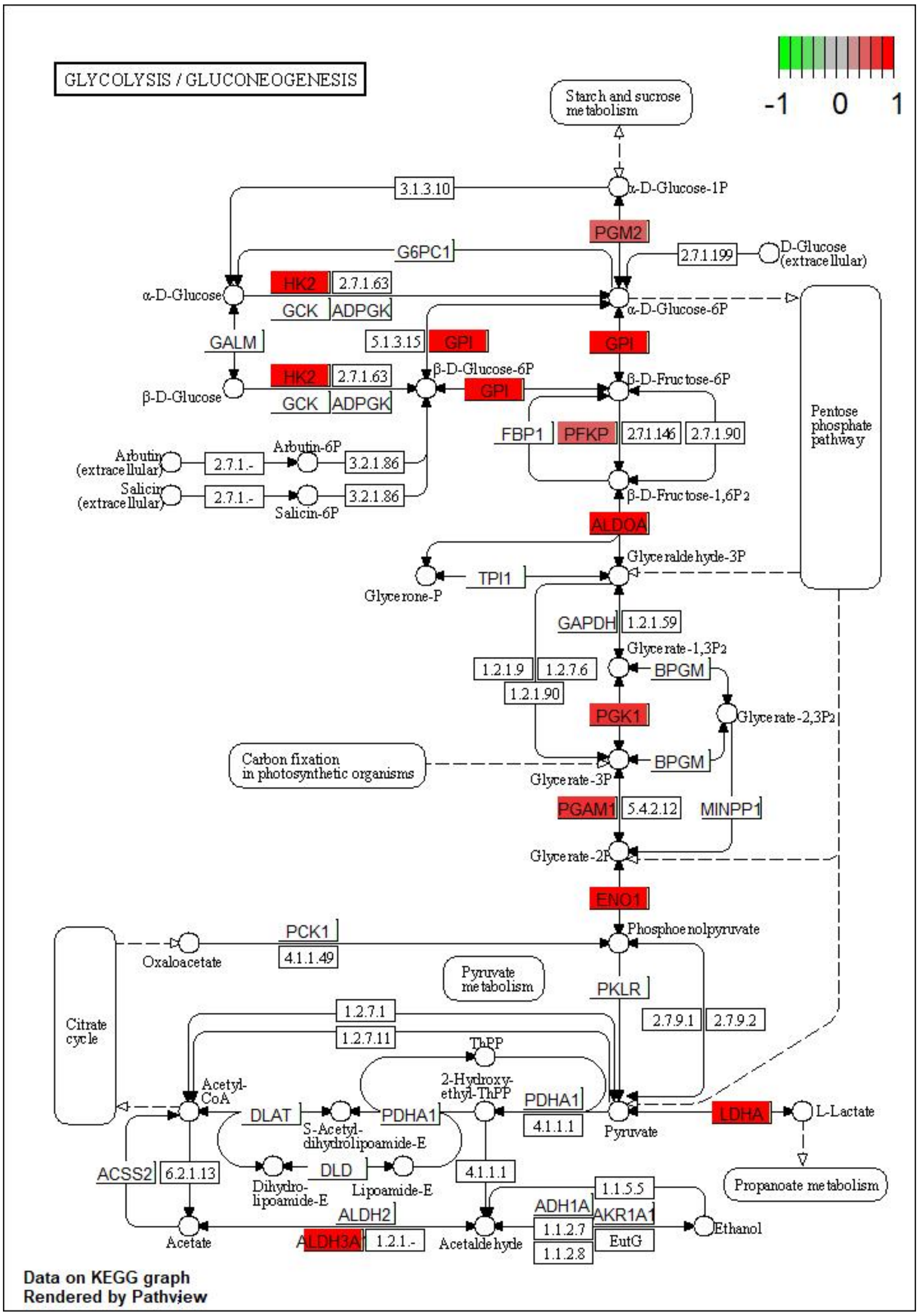

Figure: Kegg analysis of sc-RNA data by Pathview revealed alterations of Glycolysis pathway in CSC. The red box showed up-regulated genes, green box showed down-regulated genes.

S.figure Glutathione metabolism (from scRNA-sequencing data)

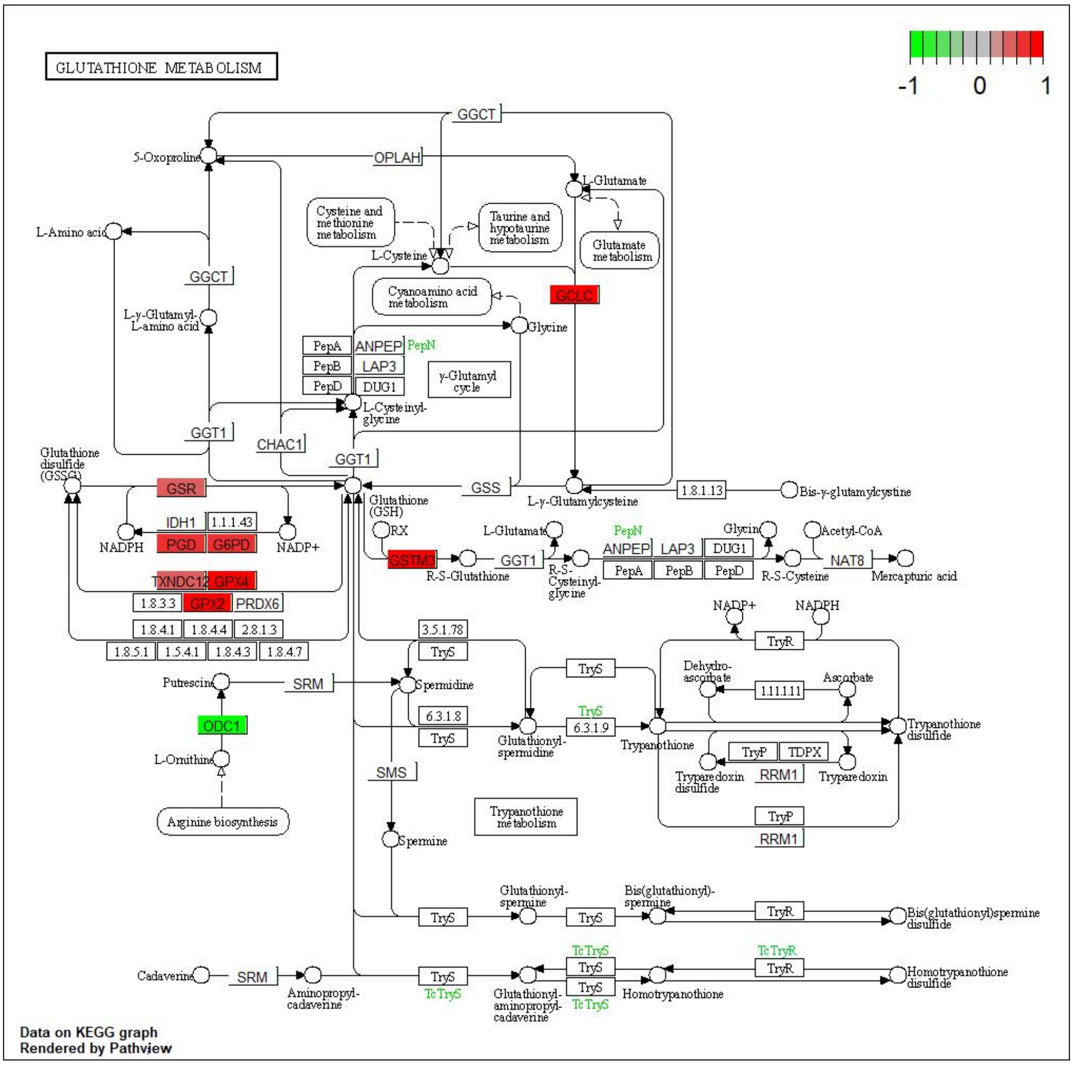

Figure: Kegg analysis of sc-RNA data by Pathview revealed alterations of Glutathione metabolism in CSC. The red box showed up-regulated genes, green box showed down-regulated genes.

S.figure Glycerophospholipid metabolism (from scRNA-sequencing data)

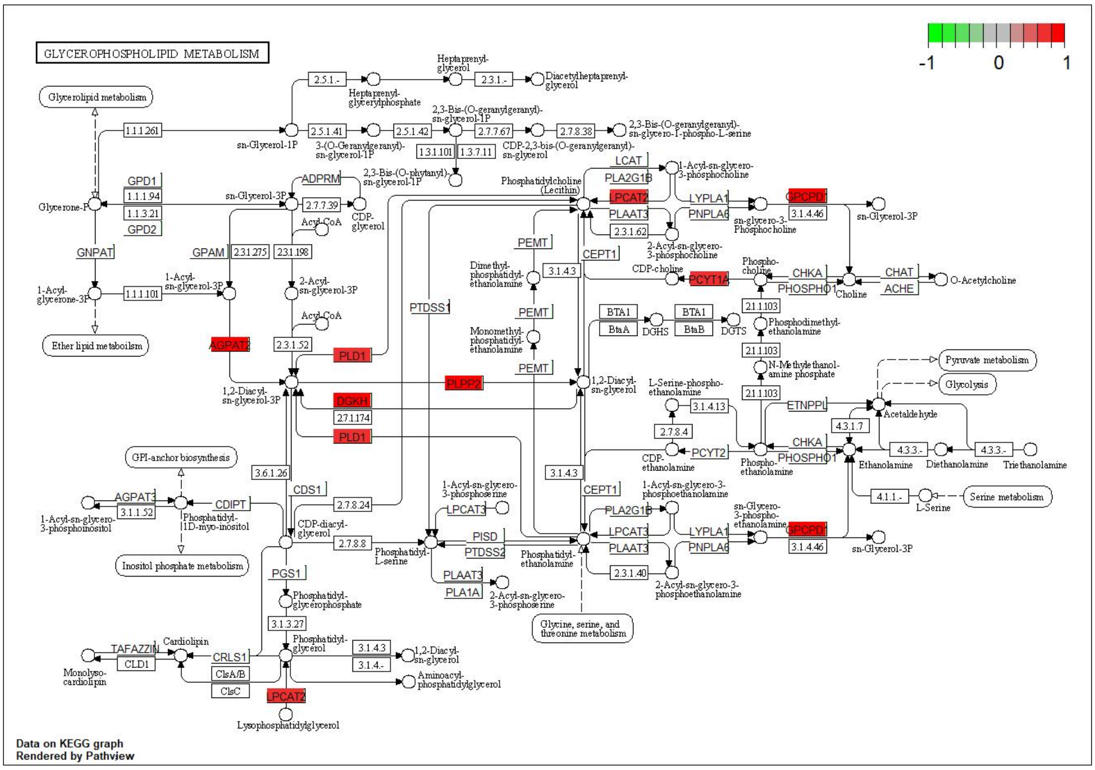

Figure: Kegg analysis of sc-RNA data by Pathview revealed alterations of Glycerophospholipid metabolism in CSC. The red box showed up-regulated genes, green box showed down-regulated genes.
