# Supplementary material for: Metabolomics, Transcriptome and Single-Cell RNA Sequencing Analysis of the Metabolic Heterogeneity between Oral Cancer Stem Cells and Differentiated Cancer Cells
Source: Cancers (Basel). 2024 Jan 5;16(2):237. doi: 10.3390/cancers16020237 (PMC10813553; doi:10.3390/cancers16020237)
Supplement: Supplementary file 1 [file cancers-16-00237-s001.zip › Supplemently method S1.pdf]

Quasi-Targeted Metabolomics - Materials and Methods

1. Metabolites Extraction

Tissue sample

Tissues (100 mg) were individually grounded with liquid nitrogen and the homogenate was resuspended with prechilled 80% methanol by well vortex. The samples were incubated on ice for 5 min and then were centrifuged at 15,000 g, 4°C for 20 min. Some of supernatant was diluted to final concentration containing 53% methanol by LC-MS grade water.Thesamples were subsequently transferred to a fresh Eppendorf tube and thenwere centrifuged at 15000 g, 4°C for 20 min. Finally, the supernatant was injected into the LC-MS/MS system analysis[1].

Liquid sample

The samples (100 μL) were placed in the EP tubes and resuspended with prechilled 80% methanol by well vortex. Then the samples were incubated on ice for 5 min and centrifuged at 15,000 g, 4°C for 20 min. Some of supernatant was diluted to final concentration containing 53% methanol by LC-MS grade water.The samples were subsequently transferred to a fresh Eppendorf tube and thenwere centrifuged at 15000 g, 4°C for 20 min. Finally, the supernatant was injected into the LC-MS/MS system analysis[2-3].

Cell or bacteria sample

The samples were placed in the EP tubes and resuspended with prechilled 80% methanol by well vortex. Then the samples were melted on ice and whirled for 30 s. After the sonification for 6 min, they werecentrifuged at 5,000 rpm, 4°C for 1 min. The supernatant was freeze-dried and dissolvedwith 10% methanol. Finally, the solution was injected into the LC-MS/MS system analysis[4-5].

Cell or bacteria culture medium sample

The samples (1 mL) were freeze-dried and resuspended with prechilled 80% methanol by well vortex. Then the samples were incubated on ice for 5 min and centrifuged at 15,000 g, 4°C for 15 min. Some of supernatant was diluted to final concentration containing 53% methanol by LC-MS grade water.The samples were subsequently transferred to a fresh Eppendorf tube and thenwere

centrifuged at 15000 g, 4°C for 15 min. Finally, the supernatant was injected into the LC-MS/MS system analysis.

2. HPLC-MS/MS Analysis

LC-MS/MS analyses were performed using an ExionLC™ AD system (SCIEX) coupled with a QTRAP® 6500+ mass spectrometer (SCIEX) in Novogene Co., Ltd. (Beijing, China). Samples were injected onto a Xselect HSS T3 (2.1×150 mm, 2.5 μm) using a 20-min linear gradient at a flow rate of 0.4 mL/min for the positive/negative polarity mode. The eluents were eluent A (0.1% Formic acid-water) and eluent B (0.1%Formic acid-acetonitrile)[6]. The solvent gradient was set as follows: 2% B, 2 min; 2-100% B, 15.0 min; 100% B, 17.0 min；100-2% B, 17.1 min；2% B, 20 min. QTRAP® 6500+ mass spectrometer was operated in positive polarity mode with Curtain Gas of 35 psi, Collision Gas of Medium, IonSpray Voltage of 5500V, Temperature of 550℃, Ion Source Gas of 1：60, Ion Source Gas of 2：60. QTRAP® 6500+ mass spectrometer was operated in negative polarity mode with Curtain Gas of 35 psi, Collision Gas of Medium, IonSpray Voltage of -4500V, Temperature of 550℃, Ion Source Gas of 1：60, Ion Source Gas of 2：60.

3. Metabolites Identification and Quantification

The detection of the experimental samples using MRM (Multiple Reaction Monitoring) were based on novogene in-house database. The Q3 were used to the metabolite quantification. The Q1, Q3, RT (retention time), DP (declustering potential) and CE (collision energy) were used to the metabolite identification. The data files generated by HPLC-MS/MS were processed using the SCIEX OS Version 1.4 to integrate and correct the peak. The main parameters were set as follows: minimum peak height, 500; signal/noise ratio, 5; gaussian smooth width, 1. The area of each peak represents the relative content of the corresponding substance.

4. Data Analysis

These metabolites were annotated using the KEGG database (http://www.genome.jp/kegg/)、HMDB database (http://www.hmdb.ca/) and Lipidmaps database (http://www.lipidmaps.org/).Principal components analysis (PCA) and Partial least squares discriminant analysis(PLS‐DA) were performed at metaX[6](a flexible and comprehensive software for processing metabolomics data).We applied univariate analysis (t-test) to calculate the statistical significance (P-value).The metabolites with VIP > 1 and P-value< 0.05 and fold change≥2 or FC

≤0.5 were considered to be differential metabolites.Volcano plots were used to filter metabolites of interest whichbased on Log2(FC) and -log10(P-value) of metabolites by ggplot2 in R language.

For clustering heat maps, the data were normalized using z-scores of the intensity areas of differential metabolites and were ploted by Pheatmap package in R language. The correlation between differential metabolites were analyzed by cor () in R language (method=pearson).Statistically significant of correlation between differential metabolites were calculated by cor.mtest() in R language. P-value < 0.05 was considered as statistically significant and correlation plots were ploted by corrplot package in R language.The functions of these metabolites and metabolic pathways were studied using the KEGG database.The metabolic pathways enrichment of differential metabolites was performed, when ratio were satisfied by x/n > y/N, metabolic pathway were considered asenrichment, when P-value of metabolic pathway < 0.05, metabolic pathway were considered as statistically significant enrichment.

Reference

[1] Want E J , Masson P , Michopoulos F , et al. Global metabolic profiling of animal and human tissues via UPLC-MS[J]. Nature Protocols, 2012, 8(1):17-32.

[2] Want E J , O"Maille G , Smith C A , et al. Solvent-Dependent Metabolite Distribution, Clustering, and Protein Extraction for Serum Profiling with Mass Spectrometry[J]. Analytical Chemistry, 2006, 78(3):743-752.

[3] BarriT ,Dragsted L O . UPLC-ESI-QTOF/MS and multivariate data analysis for blood plasma and serum metabolomics: effect of experimental artefacts and anticoagulant[J]. Analytica Chimica Acta, 2013, 768(1):118-128.

[4] Sellick C A, Hansen R, Stephens G M, et al. Metabolite extraction from suspension cultured mammalian cells for global metabolite profiling[J]. Nature Protocol, 2011, 6(8):1241-9.

[5] Yuan M, Breitkopf S B, Yang X, et al. A positive/negative ion-switching, targeted mass spectrometry-based metabolomics platform for bodily fluids, cells, and fresh and fixed tissue[J]. Nature Protocols, 2012, 7(5):872-81.

[6] Ping L, Weidong D, Peiyuan Y, et al. MRM-Ion Pair Finder: a systematic approach to transform non-targeted mode to pseudo-targeted mode for metabolomics study based on liquid chromatography-mass spectrometry[J]. analytical chemistry, 87 (2015) 5050–5055.

[7] Wen B , Mei Z , Zeng C , et al. metaX: a flexible and comprehensive software for processing metabolomics data[J]. BMC Bioinformatics, 2017, 18(1).
