# Supplementary material for: Metabolomics, Transcriptome and Single-Cell RNA Sequencing Analysis of the Metabolic Heterogeneity between Oral Cancer Stem Cells and Differentiated Cancer Cells
Source: Cancers (Basel). 2024 Jan 5;16(2):237. doi: 10.3390/cancers16020237 (PMC10813553; doi:10.3390/cancers16020237)
Supplement: Supplementary file 1 [file cancers-16-00237-s001.zip › Table S1 sample detail.pdf]

S.Table sample details

experiments	Sample type	Sample source	Biological group	Biological replication
Transcriptome sequencing	Cell lines	Cal27	Cal27_MCTS	CAL_S_1
				CAL_S_2
				CAL_S_3
			Cal27_control	CAL_A_1
				CAL_A_2
				CAL_A_3
		HSC3	HSC3_MCTS	HSC_S_1
				HSC_S_2
				HSC_S_3
			HSC3_control	HSC_A_1
				HSC_A_2
				HSC_A_3
Quasi-targeted metabolomics	Cell lines	Cal27	Cal27_MCTS	CAL27_MCTS_1
				CAL27_MCTS_2
				CAL27_MCTS_3
				CAL27_MCTS_4
				CAL27_MCTS_5
			Cal27_control	CAL27_1
				CAL27_2
				CAL27_3
				CAL27_4
				CAL27_5
		HSC3	HSC3_MCTS	HSC3_MCTS_1
				HSC3_MCTS_2
				HSC3_MCTS_3
				HSC3_MCTS_4
				HSC3_MCTS_5
			HSC3_control	HSC3_1
				HSC3_2
				HSC3_3
				HSC3_4
				HSC3_5
Single-cell RNA sequencing	Clinical samples	Public database	OSCC_01	
			OSCC_02	
			OSCC_03	
			OSCC_04	
			OSCC_05	
			OSCC_06	
